# Predictors and moderators of outcomes of HIV/STD sex risk reduction interventions in substance abuse treatment programs: a pooled analysis of two randomized controlled trials

**DOI:** 10.1186/1747-597X-9-3

**Published:** 2014-01-16

**Authors:** Paul Crits-Christoph, Robert Gallop, Jaclyn S Sadicario, Hannah M Markell, Donald A Calsyn, Wan Tang, Hua He, Xin Tu, George Woody

**Affiliations:** 1 Department of Psychiatry, University of Pennsylvania, Philadelphia, PA, USA; 2 Department of Mathematics, West Chester University, West Chester, PA, USA; 3 Department of Biostatistics and Computational Biology, University of Rochester, Rochester, NY, USA; 4 The Department of Psychiatry and Behavioral Science, University of Washington School of Medicine, Seattle, WA, USA

**Keywords:** HIV prevention intervention, Skills building, Randomized controlled trial, Predictors, Moderators

## Abstract

**Background:**

The objective of the current study was to examine predictors and moderators of response to two HIV sexual risk interventions of different content and duration for individuals in substance abuse treatment programs.

**Methods:**

Participants were recruited from community drug treatment programs participating in the National Institute on Drug Abuse Clinical Trials Network (CTN). Data were pooled from two parallel randomized controlled CTN studies (one with men and one with women) each examining the impact of a multi-session motivational and skills training program, in comparison to a single-session HIV education intervention, on the degree of reduction in unprotected sex from baseline to 3- and 6- month follow-ups. The findings were analyzed using a zero-inflated negative binomial (ZINB) model.

**Results:**

Severity of drug use (*p* < .01), gender (*p* < .001), and age (*p* < .001) were significant main effect predictors of number of unprotected sexual occasions (USOs) at follow-up in the non-zero portion of the ZINB model (men, younger participants, and those with greater severity of drug/alcohol abuse have more USOs). Monogamous relationship status (*p* < .001) and race/ethnicity (*p* < .001) were significant predictors of having at least one USO vs. none (monogamous individuals and African Americans were more likely to have at least one USO). Significant moderators of intervention effectiveness included recent sex under the influence of drugs/alcohol (*p* < .01 in non-zero portion of model), duration of abuse of primary drug (*p* < .05 in non-zero portion of model), and Hispanic ethnicity (*p* < .01 in the zero portion, *p* < .05 in the non-zero portion of model).

**Conclusion:**

These predictor and moderator findings point to ways in which patients may be selected for the different HIV sexual risk reduction interventions and suggest potential avenues for further development of the interventions for increasing their effectiveness within certain subgroups.

## Background

HIV risk reduction education is often provided in substance abuse treatment programs because of the documented association between substance abuse and HIV risk behaviors [1-5]. For example, survey studies of clinics participating in the National Institute on Drug Abuse Clinical Trials Network (NIDA CTN) have indicated that most provide HIV risk reduction education [6,7]. Typically, these consist of single 30- to 90-minute sessions delivered in group or individual formats and are limited to providing information rather than improving motivation and teaching skills (e.g., role plays, etc.).

A number of studies have examined the effectiveness of psychosocial interventions for reducing injection and sexual risks for HIV in drug users. A meta-analysis of 35 such studies concluded that, in general, there are minimal differences identified between multi-session psychosocial interventions and standard educational interventions for both drug risks (injection) and sexual risks for HIV, though both types of interventions typically result in relatively large pre-post changes in risk behaviors [8]. However, some evidence for single-gender groups was found in this review.

Two such gender-based studies of HIV sexual risk reduction were conducted in the context of the NIDA CTN [9,10]. Specifically, NIDA CTN investigators were interested in seeing if a multi-session motivational and skills training program would improve the results of the typical single-session HIV sexual risk reduction education sessions and to that end. Separate randomized controlled trials for men (CTN0018) and women (CTN0019) were conducted because the skills training components of these interventions were gender-specific, very detailed and personal.

In CTN0018, men in methadone maintenance or outpatient psychosocial treatment were randomly assigned to attend either “Real Men Are Safe” (REMAS); five sessions containing information, motivational exercises, and skills training (e.g. understanding and managing the interplay between substance use and sexual performance), or HIV education (HIV-Ed; one session containing HIV prevention information). The main outcome results of CTN0018 revealed that REMAS participants engaged in significantly fewer unprotected vaginal and anal sexual intercourse occasions (USO) during the 90 days prior to the 3- and 6-month follow-ups than HIV-Ed participants [9]. For those who completed the REMAS program, the results were even stronger, with completers reducing their number of USO by 21% from baseline to 6-month follow-up. In contrast, HIV-Ed completers increased the number of USO by 2%.

The CTN0019 study of women also yielded positive results for the 5-session safe sex skills building (SSB) intervention (e.g. use of safer sex negotiation and risky sex refusal skills) compared to the 1-session HIV education intervention [10]. A significant difference between the intervention groups in mean USOs was found over time, with both groups decreasing USO at 3 months but the SSB group maintaining this improvement at 6 months and the single-session HIV group returning to baseline USO levels. Women in SSB had 29% fewer USOs than those in the single-session HIV education group.

Although statistically significant results were evident in both trials, the overall effects were not large. In CTN0018, at 3 months the effect size (Cohen’s *d*) was 0.10 for all subjects and 0.21 for completers. In CTN0019, the intervention group difference was not significant at 3 months, while the effect size was 0.42 at 6 months. Because these were effectiveness studies conducted in community-based clinics, there was substantial sample heterogeneity that may have reduced the overall between-group effects. For example, the samples consisted of patients in both methadone and drug-free outpatient clinics; primary drugs of abuse included cocaine, heroin, alcohol, and methamphetamine; severity of drug use varied, as did the tendency to engage in sex under the influence of drugs/alcohol; patients differed on age (about half under 40 and half over 40 years of age), race/ethnicity (about 60% European American; 40% minority), education (about 1/3 over 12 years), and monogamy status (about half currently monogamous). In fact, the effectiveness of REMAS in the CTN0018 study was found to differ significantly based on treatment setting (drug-free vs. methadone maintenance) [11]. However, no attention has been directed at determining whether the heterogeneity of outcomes in the CTN0018 and CTN0019 studies can be attributed to patient characteristics.

Investigating moderators of intervention effectiveness has important clinical and financial implications. Although in both CTN0018 and CTN0019 the 5-session skills building intervention was more effective than a single educational session, administering a 5-session HIV intervention to all patients would raise issues of cost and patient interest/compliance. Identifying moderators of intervention effectiveness would potentially allow for matching strategy where the 5-session REMAS and SSB interventions could be targeted at those most likely to benefit. To date, there has been limited exploration of moderator variables in the CTN0018 and CTN0019 studies, largely because statistical power was limited for investigating such relationships within each study. However, preliminary analyses within the CTN0018 study data has suggested that European Americans benefitted more from the REMAS intervention than African Americans in their rates of condom use, and that none of the Hispanic men who attended the REMAS intervention were frequently using condoms with their casual sex partners [12]. These results suggested a potential differential intervention effect for European Americans, compared to African American and Hispanic men and has led to a new version of the REMAS intervention with adaptations for using the intervention with African American and Hispanic men.

In addition to the examination of moderators, it is also useful to identify potential predictors of outcome, i.e., patient variables that are associated with outcome across both intervention groups. Information on predictors might be useful in the re-design of both types of interventions used in the CTN0018/CTN0019 studies. Alternatively, information on predictors of outcome might suggest that HIV risk reduction for those not expected to benefit from either intervention should be addressed via other means (e.g., individual counseling; referral to psychotherapist). The goal of the current study was to examine potential predictors (main effects) and moderators (interactions between predictors and intervention group) of outcomes within a pooled database of CTN0018 and CTN0019 study data. The pooled database allowed for greater statistical power for testing predictor/moderator variables and also allowed for examination of whether any moderator effects varied by gender. Two potential primary predictor/moderator variables were identified a priori: 1) severity of drug use, and 2) if the patient recently engaged in sex under the influence of drugs/alcohol. Heavy alcohol/drug use [3,13-17] and engaging in sex under the influence of alcohol or drugs [4,5,18-21] are viewed as important risk factors for HIV. We hypothesized that individuals with a greater severity of drug use, and those who engage in sex under the influence, would have relatively worse outcomes in both intervention conditions because high levels of drug severity and sex under the influence will continue to drive risky sex behaviors following the intervention. However, we also hypothesize that these variables will be moderators.

The more intensive intervention, with a focus on sex under the influence and more time to repeatedly address patient issues, has greater potential to modify drug severity and sex under the influence, leading to better outcomes in the 5-session intervention for those with these risk factors compared to the 1-session intervention. Testing for these moderators will provide information that is useful for dissemination of the study intervention.

Secondary predictor/moderator variables also of interest included: 1) whether or not the patient was currently in a monogamous relationship (this variable was used as a covariate in the primary outcome reports [9,10]); 2) duration of use of the primary substance; 3) age; 4) gender; and 5) specific racial/ethnicity groups. Patients hypothesized to be at greatest risk and therefore more likely to benefit from the study intervention included those not in a monogamous relationship, long-term users, and younger patients. Gender was explored as a potential moderator due to existing research, which indicates that gender-specific HIV-prevention interventions are more successful [22,23]. Existing research also laid the foundation for examining race/ethnicity as a predictor and moderator. Studies [24] have indicated that minorities have relatively worse outcomes following HIV-prevention interventions. Furthermore, preliminary examination (without statistical testing) of the CTN0018 data [12] suggested that the more intensive 5-session intervention was relatively less effective for African Americans and Hispanics compared to European Americans, but no such difference was evident with the 1-session intervention (i.e., a moderator effect).

## Methods

### Overview

As mentioned, the CTN0018 and CTN0019 studies had identical designs, with the same assessments and the same comparison condition (single-session HIV education group), allowing the two datasets to be combined. In both studies, the primary outcome was a count of unprotected vaginal and anal sexual intercourse occasions over the past 90 days, measured at baseline and at 3- and 6-month follow up assessments. Detailed descriptions of the study methods are given in in the primary outcome reports from these studies [9,10].

### Participants

The CTN0018 study was conducted in 14 sites, and the CTN0019 study in 12 sites across the United States; half of the sites were methadone clinics and half psychosocial outpatient clinics. For both studies, inclusion criteria were similar: 1) adults 18 years and older in drug abuse treatment; 2) self-report of engaging in unprotected vaginal or anal intercourse during the past 6 months; 3) agreeable to being randomly assigned to the study intervention groups; 4) able to speak and understand English. The primary exclusion criteria in both studies were: 1) observable, gross mental status impairment; 2) attempting to get pregnant (female study) or having a primary sexual partner who is currently planning on attempting to get pregnant (male study); 3) current treatment episode of methadone maintenance is less than 30 days. Participants were recruited through posters displayed in clinics, announcements at group therapy meetings, clinic “open houses” designed to introduce the study to clinic patients, and referrals by clinic counselors and/or staff. Combining the two studies, a total of 1,105 participants were randomized (541 to the 5-session intervention; 564 to the 1-session intervention).

### Procedures

#### Assessments

After determination of eligibility and consent, participants completed a 2- to 3-hour baseline assessment. This included assessment of the primary outcome measure, which was obtained from the Sexual Risk Behavior Assessment Schedule (SERBAS) [25,26] in CTN0019. In the CTN0018 study, the SERBAS questions were embedded in a larger Sexual Behavior Inventory (SBI) that also included questions adapted from the Sex and Drug Abuse Relationship Interview [20]. The SERBAS was administered at baseline, month 3, and month 6 using the audio computer-assisted self-interviewing method. Items included questions regarding: 1) frequency of unprotected vaginal, anal, oral sex by partner type (main versus casual); 2) number, gender, and HIV serostatus of partners (if known); 3) the percentage of times sex occurred under the influence of drugs or alcohol over the 90 days prior to filling out the measure. The primary outcome measure in both studies was a count of the number of unprotected sexual intercourse occasions (USOs) in the past 90 days. This measure was calculated by adding the total number of vaginal and anal intercourse occasions and subtracting the number of those sexual acts for which the participant reported the use of either a male or female condom. Participants were also administered a simplified version of the Addiction Severity Index (ASI) [27], which included information on demographics, drug and alcohol use, and related problem areas. From the ASI we extracted variables to be examined as predictors/moderators, including severity of current drug use (ASI Drug Use composite), duration of drug use, age, gender, and race/ethnicity. With race/ethnicity, our interest was in the impact of specific racial/ethnic group membership on outcome, so race/ethnicity was coded in terms of three contrast variables: (1) non-Hispanic European Americans vs. all others, (2) non-Hispanic African Americans vs. all others, and (3) Hispanics vs. all others. In addition, exploratory analyses examined amount of use of specific drugs (alcohol, cocaine, opioids, amphetamines, cannabis), obtained from the ASI. From the SERBAS, we extracted information about whether or not the patient engaged (recently) in sex under the influence of drugs/alcohol and whether or not the patient was currently in a monogamous relationship.

Monogamy status was included as a covariate in the primary analyses for both the CTN0018 [9] and CTN0019 [10] studies because of the reported association between primary relationships and reduced levels of condom use [28-30]. In CTN0019, monogamy status was based on the woman’s self-report of whether she considers any male partner to be her “main” partner and whether or not she reports any other (male or female) partners, taken from the SERBAS. In CTN0018, monogamy status was derived from similar questions in the SBI.

After completion of the baseline assessment, eligible participants were placed in a holding cohort awaiting randomization. Once there were eight participants in the cohort or 3 weeks had passed (whichever came first), the cohort was randomized to one of the two interventions. Randomization was delayed if there were less than three participants in a cohort at the end of 3 weeks until there were at least three participants. Every effort was made to keep the research staff assigned to complete follow-up assessments unaware of the intervention assignment for each participant. In CTN0018, the blinding procedures were moderately effective at 3 months (research assistants reported knowing the intervention assignment of 41.0% of participants) and somewhat more effective at the 6-month follow-up (22.8% of intervention assignments were known). In CTN0019, blinding procedures were more effective: 17.5% at 3 months, and 13.2% at 6 months, were correctly identified. Because the primary outcome measure was obtained by computer interview, the impact of unblinded research assistants was minimized.

#### Interventions

The REMAS intervention used in the CTN0018 study and the SSB intervention used in the CTN0019 study were workshops of five, 90-minute group sessions. To supplement informational lectures, there was liberal use of role-plays, peer group discussions, and self-assessment motivational exercises, with a nearly equal focus on information delivery and skill building and a somewhat smaller focus on motivation. There was also discussion of the combining of sexual behavior and drug use and, in the SSB intervention, women’s negotiation skills around safer sex and safeguards against the risk of partner abuse as the potential result of assertiveness around safer sex.

The HIV-Ed group used in both studies was intended to represent a standardized treatment-as-usual intervention that would be appropriate for groups of men, women, or mixed men and women. This one-session (60 minutes) intervention consisted of selected educational material covering: HIV/AIDS definitions, transmission, testing and counseling, treatment, and prevention. Counselors delivering this intervention used a didactic presentation style and question-and-answer format along with flip chart visual materials and handouts.

The two group interventions were delivered by male (for CTN0018) and female (for CTN0019) counselors already employed in the study clinics. Groups were conducted by co-leaders who shared responsibility for delivery of the treatment. The treatment counselors received approximately 30 hours of training in conducting both interventions. Treatment manuals were used for training for all of the interventions. A clinical supervisor at each site was also trained in conducting the interventions and provided on-site supervision to the counselors.

During the training sessions, counselors and supervisors practiced intervention skills. Supervisors also practiced supervision skills and rated counselors on adherence to the manuals while they practiced. Counselors and supervisors were certified as sufficiently competent at delivering the interventions if they demonstrated at least adequate proficiency on mock exercises.

During the actual study, all counselors and supervisors participated in bi-weekly conference calls with study lead trainers to problem-solve difficult clinical situations and to share intervention experiences. In addition, local supervisors conducted weekly supervision sessions at each site. All sessions were audiotaped and local supervisors rated about half of all tapes in CTN0018 and 150 tapes in CTN0019 for adherence. For CTN0018, 92.9% of the 5-session intervention tapes, and 91.4% of the 1-session intervention tapes, were rated as meeting fidelity criteria. For CTN0019, rates of adherence were 80.2% for the 5-session intervention and 87.2% for the 1-session intervention. Corrective feedback was provided by the supervisor when adherence ratings fell below adequate proficiency levels.

### Statistical analysis

The analyses were conducted using all randomized participants with baseline and at least one 3- or 6-month follow-up outcome assessment. Within this constraint, the data analytic approach was a longitudinal model using all available month 3 and month 6 scores. The longitudinal statistical models estimated the population average of month 3 and 6 scores (not the slopes). To account for excess zeros in the dependent variable, in the original efficacy papers from CTN0018 [9] and CTN0019 [10], different data analytic approaches were taken, with the CTN018 study [9] using a zero-inflated Poisson (ZIP) model and the CTN0019 study [10] using a generalized mixed-effects model with a Poisson distribution and logarithmic link function. A subsequent article [31] using the CTN0019 data showed that a zero-inflated negative binomial (ZINB) model [32] fit the data best. We conducted preliminary analyses on the pooled CTN0018/CTN0019 dataset to examine whether ZIP, ZINB, or Poisson regression models provided the best fit. The comparison of the goodness of fit used Vuong’s [33] statistic and the significance testing provided by Khoshgoftaar et al. [34]. We also graphically examined the fit of the models to the data. The results of these analyses showed clearly that a ZINB model fit the data significantly better than ZIP (*p* < .0001), Negative Binomial (*p* < 0.0001), or Poisson regression (*p* < .0001) models. A ZINB analysis yields results in terms of two components of outcome: (1) a “zero” component that examines the dependent variable in terms of zero vs. non-zero scores, and (2) a “count” portion that examines only those individuals with a non-zero score and uses the full distribution of count responses (i.e., 1, 2, 3, 4, ….).

Using ZINB models, we separately examined the primary and secondary baseline variables as main effect predictors and moderators (treatment by predictor interaction) of the month 3 and 6 outcomes in the longitudinal models. Time was included in the statistical models, as were the following covariates: site, baseline level of the dependent variable, and study (gender). We also tested whether any moderation effects varied by gender (study) with a 3-way interaction. Analyses proceeded hierarchically, with main effects (predictors) evaluated without interaction terms, followed by models that included main effects and a two-way interaction term (moderator effects), followed by models that included main effects, a two-way interaction, and a 3-way interaction (moderators by gender). In addition to each predictor/moderator tested separately, we conducted a multivariable test with all (non-redundant) variables in the model. The SAS software and the NLMIXED procedure, specifically modified to account for the ZINB structure as well as accommodate the clustering due to the repeated measures, were used for the analysis.

## Results

### Study sample

Demographics for those randomized and the analysis sample are presented in Table 1. In general, the randomized samples were about 65% European American, 26% African American, and 6% Hispanic. The average age was about 39 years, and about 43% had never been married. Of the 1,105 subjects randomized, analyses were conducted on 824 who had either a 3- or 6-month outcome. There were no significant differences between the two intervention groups on subject characteristics shown in Table 1 for either the randomized sample or analysis sample. A complete analysis of attrition/missing data in these studies (differences between completers and drop-outs; impact of drop-out on outcome) is provided in the original study reports [9,10].

**Table 1:** Participant characteristics for combined CTN0018/CTN0019 sample

	**Randomized sample (N = 1105)**		**Analysis sample (N = 824)**	
	**5 session**	**1 session**	**5 session**	**1 session**
	**Skills training**	**HIV-Ed**	**Skills training**	**HIV-Ed**
	**(N = 541)**	**(N = 564)**	**(N = 395)**	**(N = 429)**
	Mean and (Standard Deviation)			
Age	38.8 (10.2)	39.0 (9.4)	39.8 (10.3)	39.8 (9.4)
Education in years	12.1 (1.9)	12.2 (2.0)	12.0 (1.9)	12.2 (2.0)
Monthly net income	$344.8 (873.6)	$340.1 (970.2)	$326.7 (849.9)	$333.3 (936.4)
	Percent			
Race/Ethnicity				
European American	64.9	64.9	62.3	64.8
African American	25.0	28.4	25.6	28.2
Hispanic	7.4	5.1	9.1	6.00
Am. Indian	1.7	0.9	1.5	0.9
Asian	0.6	0.5	0.8	0.5
Other	0.6	0.2	0.0	0.0
Marital Status				
Never married	42.0	43.9	41.3	43.1
Married	21.4	17.8	22.8	18.2
Divorced	22.9	21.1	22.0	20.3
Separated	9.6	13.5	9.4	14.00
Remarried	0.4	0.7	0.5	0.9
Widowed	3.7	3.0	3.5	3.5
Program Type				
Methadone	48.6	49.7	60.00	59.2
Psychosocial	51.4	50.4	43.0	40.8
Gender				
Women	46.0	47.0	44.8	48.3

*Note*. The 5 Session Skills Training and 1 session HIV-Education groups are not statistically different from each other (*p* > .05) for either the randomized or analysis samples.

### Evaluating heteroscedasticity

To determine the appropriateness of the analyses with the ZINB models, we conducted tests for heteroscedasticity. These analyses focused on the count portion of the model. For the zero portion of the model, differences in outcome variability within levels of each predictor variable are directly linked to the statistical significance of the predictor in discriminating between no versus any unprotected occurrences. However, with respect to the count portion of the ZINB model, difference in variances in the outcome as a function of the levels of a predictor variable may be indicative of differential restricted ranges producing or masking moderation effects. To assess for such potential heteroscedasticity, we perform Levene’s homeogeneity of variance test on the number of unprotected sexual occasions across the levels of the respective predictors. For the continuous predictors (ASI Drug Use Composite, age, and duration of drug problems) we used quartile splits to produce four categories. No variables showed evidence of violation of homogeneity of variance (all *p’s* > .10).

### Predictors

In single predictor analyses, controlling for intervention group and baseline scores on the dependent variable, severity of drug use (ASI Drug Use composite) was a significant main effect predictor of number of unprotected sexual occasions, given any such association (i.e., the count portion of the ZINB analysis) (Table 2). Across patients, an increase of 1 standard deviation on the ASI Drug Use composite was associated with a 17.1% increase in the number of unprotected sexual occasions (averaging over the 3- and 6-month assessments; controlling for baseline levels of unprotected sexual occasions). To understand what type of drug use was driving this effect, we explored the relation of days using each specific type of the most common drugs of abuse (cannabis, alcohol, cocaine, opioids, amphetamines, polydrug use) in the past month to outcome in the count portion of the ZINB analyses. Significant effects were evident for two of these individual predictors: alcohol (*t* = -2.84, *DF* = 1447, *p* < .01) and polydrug use (*t* = 3.30, *DF* = 1447, *p* < .01), such that, across patients, an increase of 1 standard deviation in alcohol use corresponded to a 13.1% decrease in the number of unprotected sexual occasions, whereas for polydrug use an increase of 1 standard deviation in use corresponded to an 18.8% increase in the number of unprotected sexual occasions.

**Table 2:** Parameter estimates (PE) and associated t-values for predictors and moderators in relation to number of unprotected sexual occasions

**Baseline variable**	**Predictor (Main effect)**				**Moderators (Intervention group by predictor)**			
	**Zero**		**Count**		**Zero**		**Count**	
**Main Hypotheses**	t	PE	t	PE	t	PE	t	PE
Severity of Current Primary Drug Use								
ASI Drug Use Composite	-1.21	-1.24	2.91	1.17**	-.41	-.65	-.60	-.38
Recent Sex Under the Influence	.31	.08	-.01	-.00	-1.14	-.54	2.94	.57**
**Secondary Hypotheses**								
Duration of Drug Problem	-.81	-.01	-1.44	-.01	.05	.00	1.98	.02*
In monogamous relationship?	4.29	1.06***	-1.22	-.11	1.69	.83	.83	.15
Gender (men = 1; women = 0)	.06	.01	2.52	.26*	-.52	-.27	.97	.18
Age	-.44	-.01	-4.13	-.02***	.54	.83	-1.19	-.01
Race/Ethnicity								
European American, non-Hispanic vs.	-3.43	-.85***	1.25	.12	-.44	-.20	-.66	-.12
All others								
African American, non-Hispanic vs.	2.87	.93***	-2.83	-.32**	-.59	-.35	.05	.01
All others								
Hispanic vs. all others	1.27	.45	.03	.05	2.59	1.60**	2.18	.53*
**Main Hypotheses**								
Severity of Current Primary Drug Use								
ASI Drug Use Composite	.14	.17	1.68	.73	-1.17	-2.83	-.92	-.64
Recent Sex Under the Influence	.52	.15	-.52	-.05	-1.14	.82	2.67	.56**
**Secondary Hypotheses**								
Duration of Drug Problem	-.71	-.01	-.25	-.00	1.15	.04	2.17	.02*
In monogamous relationship?	4.06	1.14***	-1.19	-.12	1.00	.65	.93	.18
Gender (men = 1; women = 0)	-.57	-.19	2.92	.34**	1.62	-1.06	.73	.15
Age	-1.06	-.02	-2.07	-.01*	-1.87	-.08	-.81	-.01
Race/Ethnicity								
African American, non-	2.38	.93*	-1.35	-.17				
Hispanic vs. all others								
Hispanic vs. all others					3.00	2.45**	2.05	.53*

*Note*. Parameter estimates and associated t-values from ZINB analyses are given. For univariable tests, the DF for the t-values is 1454, except for Recent Sex Under the Influence, for which the DF was 1365. For multivariate tests, the DF for each t-value is 1353. The parameter estimates for the zero column corresponds to the log-odds coefficients for a unit increase for a given predictor for any unprotected occurrence. Positive coefficients correspond to higher prevalence of occurrences per unit increase in the given predictor; a negative coefficient corresponds to lower prevalence of occurrences per unit in the given predictor. The parameter estimates for the count column corresponds to the log of the multiplicative change in the average number of unprotected occurrence per unit increase in the given predictor. Positive coefficients correspond to an increase in the number of unprotected occurrences per unit increase in the given predictor; a negative coefficient corresponds to a decrease in the number of occurrences per unit increase for the given predictor. Main effects (predictors) are evaluated without interaction terms in the models. Moderators (intervention by predictor) are evaluated with relevant main effects in the model. Outcome is number of unprotected sexual occasions at 3 and 6 months. Analysis is a longitudinal model focusing on estimating the population average over the 3 and 6 month outcome assessments. Analyses are controlling for site, study (gender), and baseline number of unprotected sexual occasions. Sample size is 824. *p < .05; **p < .01 ***p < .001.

As expected, being in a monogamous relationship was significantly associated with outcome across the 3- and 6-month assessments (Table 2). However, the association was evident only with the zero component in the ZINB analysis. Those individuals in monogamous relationships were more likely to have had occasions of unprotected sex (55.8% of non-monogamous subjects vs. 71.4% of monogamous subjects had occasions of unprotected sex; Odds ratio = 1.88).

Race/ethnicity was a significant predictor in the zero portion of the ZINB analyses across the combined sample in single predictor analyses for both European American vs. others and for African Americans vs. others (Table 2). Non-European Americans (compared to European Americans) and African Americans (compared to all others) were more likely to have at least one occasion of unprotected sex (these two effects are redundant, given that no effect was evident for Hispanics). The Odds Ratio for occurrence of unprotected contact was .427 for European Americans compared to non-European Americans and 2.537 for African Americans compared to non-African Americans. Race/ethnicity (non-Hispanic/African American vs. all others) was also significant in the count portion of the ZINB (Table 2). In this case, given non-zero unprotected sex occasions, African American individuals were less likely to have more of such occasions compared to non-African Americans. Among participants who had any unprotected sex, the median number of unprotected occasions was 13.5 for non-African Americans and 9 for African Americans (averaging over the 3- and 6-month scores).

Gender and age also showed highly significant associations with the count portion of the ZINB analysis in single predictor variable analyses (Table 2). Among participants who had any non-protected sex, the median number of unprotected sexual occasions was 10 for women and 14 for men (averaging over the 3- and 6-month scores). Interpretation of the regression coefficient from the ZINB model indicates, on-average there was a 17.3% increase in the in the number of unprotected sexual occasions for men compared to women. For age, older individuals were found to engage in a smaller number of unprotected occasions than their younger counterparts, such that there was an 18.3% decrease in the number of unprotected sexual occasions for each standard deviation increase in age.

A multivariable model was conducted incorporating all of the main effect predictors into the same model (though only African American vs. others was used as a race/ethnicity predictor given the redundancy among the race/ethnicity variables). In this multivariable model, the two significant single variable predictors (in monogamous relationship and race) for the zero portion of the ZINB analysis remained statistically significant (Table 2). For the count portion of the ZINB analyses, gender and age also remained as statistically significant in the multivariable model (Table 2). However, the ASI Drug Use composite and race were no longer significant predictors in the count portion of the ZINB multivariable model. In this sample, race (African American vs. others) was highly associated with age and the ASI Drug Use composite (African Americans were on average 5 years older than non-African Americans and had higher ASI Drug Use composite scores). Consequently, incorporating the ASI Drug Use composite, age, and race in the same model reduced the associations of all three of these variables with outcome (Table 2).

### Moderators

Of the primary variables, recent sex under the influence of drugs/alcohol was found to be a significant moderator of intervention effects (Table 2). Among those who engaged in at least one unprotected sexual occasion and did not engage in sex under the influence, the median number of unprotected sexual occasions was 12 for the single-session intervention group and 10 for the gender-specific 5-session skills group (averaging over the 3- and 6-month scores). For those who did engage in sex under the influence, the median number of unprotected sexual occasions was 15 for both intervention groups (averaging over the 3- and 6-month scores). Thus, for both groups, there were a greater number of unprotected sexual occasions when individuals were under the influence but a larger reduction in unprotected sexual occasions among those who abstained from sex under the influence for the 5-session gender-specific skills training intervention group compared to the single-session intervention group.

Among the secondary variables, duration of abusing the primary drug was a significant moderator of intervention effects in the count (non-zero) portion of the ZINB analysis (Table 2). Within the single-session HIV education group, a one-standard deviation increase in years of use of the primary drug corresponded to a 12.8% reduction in the expected number of unprotected sexual occasions over the 3- and 6-month follow-ups. Within the gender-specific skills training group, a one-standard deviation increase in years of use if the primary drug corresponded to a 3.9% increase in the expected number of unprotected sexual occasions. We divided years of use of primary drug at the median (12 years) to understand this effect. We also explored whether a quadratic interaction term (intervention by the square of duration of primary drug) fit better, but this effect was non-significant and therefore splitting at the median was a reasonable way to illustrate the effect. For the single-session intervention, the median number of occasions of unprotected sex across the 3- and 6-month follow-ups was 15 for individuals with relatively lower duration of primary drug use and 14 for individuals with relatively higher duration of primary drug use. For the 5-session intervention, the comparable numbers were 15 and 12. Thus, the 5-session intervention was particularly effective for those with a high duration of use of their primary drug.

Hispanic ethnicity (vs. all others) was also a significant moderator. The effect was evident for both the zero and non-zero portions of the ZINB analysis (Table 2). Among non-Hispanics, there was a post-intervention prevalence rate for unprotected sexual occasions of 67.1% for the single session group compared to 61.8% for the gender-specific skills intervention. Among Hispanics, prevalence rates for unprotected sexual occasions were 60.0% and 71.3% for the single session and gender-specific skills intervention effect, respectively, across the 3- and 6-month assessments. For those who engaged in at least one unprotected sexual occasion and were non-Hispanic, the median number of unprotected sexual occasions was 15 for the single-session HIV education group and 12 for gender-specific 5-session skills group (averaging over the 3- and 6-month scores). In contrast, for Hispanics, the median number of unprotected sexual occasions was 16 for the single-session group and 20 for gender-specific 5-session skills group (averaging over the 3- and 6-month scores). Thus, for both the zero and non-zero portion of the model, there were relatively better outcomes for the gender-specific skills group compared to the single-session HIV education group among non-Hispanics but relatively worse outcomes for the gender-specific skills group compared to the single-session HIV education group among the Hispanics.

All three of the moderators in the count portion of the ZINB analysis (sex under the influence; duration of drug problem; Hispanic ethnicity) remained statistically significant in a multivariable model (Table 2). No intervention moderator effects varied significantly by gender.

## Discussion

The results of the analyses presented here suggest that the relative degree of reduction in unprotected sexual occasions following both a single HIV education group session and a 5-session gender-specific skills training group were dependent on certain patient characteristics. As expected, those in monogamous relationships had reduced use of condoms. This has been found in previous research [35-37]. It has been suggested that having unprotected sex with a committed relationship partner who has not been tested for HIV may be a major and unrecognized source of HIV risk [37], a situation that may be particularly true in a high-risk population such as substance abusers. HIV sex risk interventions may need to be altered to place greater emphasis on risks even in monogamous relationships. One qualification of this conclusion, however, is that the effect found here for being in a monogamous relationship was significant only for the logistic portion of the ZINB analysis.

Relatively poorer intervention outcomes were evident for younger participants and men, controlling for other predictor variables. The effect sizes for these main effect predictor findings were all moderate in size [38], and potentially provide some guidance to clinicians about what types of individuals are at greatest risk for unprotected sex. Such individuals might be candidates for extra attention in group sessions, review of such issues in individual counseling sessions, or referral for further interventions. There were complex findings about the possible association between African American race and unprotected sex occasions. African Americans (compared to non-African Americans) were more likely, following either intervention, to engage in unprotected sex at least once. However, among those with at least one unprotected sex occasions, African Americans had fewer unprotected sex occasions (compared to non-African Americans). Since African American heterosexual women (and African American men who have sex with men), represent disproportionately large proportions of people with HIV in these risk groups [39], future research is essential to better understand the reason for this, and to tailor interventions to decrease this disparity.

The moderator results reported here provide information on the relative benefits of the 1-session versus 5-session interventions also depend on certain patient characteristics. Most notably, Hispanic individuals did relatively more poorly in the 5-session intervention than in the 1-session intervention. This finding is consistent with the preliminary (not tested statistically) suggestion from within the CTN0018 study of less effectiveness for Hispanic individuals [12] and raises the possibility that the 5-session intervention may not have addressed culturally specific issues, or used the most culturally relevant examples in the didactic elements of the intervention. These findings therefore support the development of culturally adapted versions of the 5-session intervention [40]. The primary modifications of the intervention were to add modules that addressed a stronger focus on understanding how each man’s cultural and socialization experiences about sex contribute to his past and current sexual behavior. However, further research designed to understand the exact mechanism through which Hispanic ethnicity is associated with relatively poorer outcomes of the 5-session skills building intervention may be needed so that any further intervention development steps are properly targeted to the relevant causal variables. Such further research is particularly indicated given that the moderator effects found here were only in the small to moderate range using the descriptors provided by Rosenthal [38].

An additional moderator effect occurred for recent sex under the influence. For those who engaged in sex under the influence, outcomes were relatively poor for both intervention groups. Among those who did not engage in sex under the influence, the 5-session intervention group had slightly more positive outcomes than the 1-session group. This finding is surprising given the attention paid to the topic of sex under the influence in the 5-session intervention. It should be noted, however, that in this sample, no causative link between sex under the influence of drugs or alcohol and sexual risk behavior was evident [41]. The reasons for the lack of causative link are unclear, but it may be that such a link is difficult to detect within a sample of individuals currently receiving treatment in substance abuse treatment facilities. Thus, at least in this high risk sample, the need to reduce sex under the influence in order to increase safe sex was not apparent. However, it is possible that in other types of samples it would be important to achieve greater reduction in occasions of sex under the influence. Further intervention development work might be indicated to achieve this goal.

The fact that the 5-session intervention was particularly effective, relative to the 1-session intervention, for those individuals who had a long duration of primary drug use is notable. This finding provides some justification for the added expense and effort of clinical implementation of the 5-session intervention, at least for a subgroup of individuals receiving drug abuse treatment services in community agencies.

The current study found that age, severity of drug use, gender, monogamous relationship status, and race predicted degree of reduction in risky sexual behaviors in substance users. Other studies have obtained similar results for this population (treatment-seeking substance users) with regard to substance use outcomes, finding that age [42-45]; race [46-48]; and severity of use [49,50] were each predictors of substance use outcome. Contrary to risky sex behavior outcomes; studies that have examined gender [51,52] and relationship status [53] as predictors of substance use outcomes yield inconsistent and non-significant findings, respectively. Thus, the finding for gender and relationship status found in the current study may be specific to the interventions and outcomes used herein. However, our findings for age, race, and severity of use may reflect a broader tendency for substance users to be non-compliant and/or non-responsive to a range of treatments on a range of outcomes. Further research is needed to clarify the extent to which the predictors and moderators found here are specific to the interventions and outcomes examined here. Regardless of their specificity, the present findings contribute to the potential clinical usefulness of such predictors/moderators and alerts investigators to their relevance for research designs in regard to choice of covariates when examining these interventions.

Although the data presented here provide some empirical guidance for making individual treatment prescriptions for the 5- and 1-session interventions evaluated in the CTN0018 and CTN0019 studies, additional research is needed to have a broader understanding of which HIV risk reductions interventions work best for distinct subgroups of substance users. Our analyses were restricted to a limited set of primary and secondary potential predictors/moderators. Other variables, not examined here, might also be important predictors/moderators. For example, some individuals who are less comfortable in a group setting may be relatively poor candidates for a 5-session group intervention. Interpersonal variables may be relevant to who participates the most during group sessions and therefore benefits the most from a group intervention. Aspects of individual’s sexual history and preferences may also be associated with degree of sex risk reduction evident following the 5- and 1-session interventions. Qualitative research strategies might be a particularly useful way to obtain some further insights about the range of variables that might determine who benefits the most from different HIV risk reduction interventions.

It is also important to put the findings from the current study into the context of different approaches to intervention science. As mentioned, the predictor and moderator effects found here might prompt investigators to develop new, adapted versions of their interventions. In fact, as mentioned, an adapted version of the 5-session intervention for men has already been developed [40]. However, developing a large number of adapted versions (e.g., cultural adaptation; age-related adaptation; adaptations based on gender, sexual history, and severity of use) may not be feasible from an intervention development point of view. In addition, clinicians may find that the availability of so many different versions is confusing. Alternatively, a sequential strategy can be used in which non-responders to one form of intervention receive a second, different form of intervention. At this point in time, both approaches can be pursued until it is clearer which direction is yielding more useful data.

Several limitations of the original CTN0018 and CTN0019 studies are important to keep in mind when evaluating the results of the current predictor/moderator analyses. Both studies were conducted in a variety of settings and had few exclusion criteria, but there are limits on the generalizability of any results from the data based on a number of factors, including self-referral to the study, age, type of substance of abuse, psychiatric and substance abuse diagnosis, and sexual history (i.e., women who had not had sex with a man in the past 6 months were excluded from the CTN0019 study). Another limitation is that participating counselors received 30 hours of special training in the 5-session intervention. Less training, as would be common if this 5-session intervention was implemented clinically, might yield different results than found here. An important limitation in comparing the two interventions is the difference in duration of the interventions (1 vs. 5 sessions). It may be that five sessions of standard HIV counseling would achieve comparable results to the 5-session skills building intervention.

In addition to the above limitations of the study designs, there are limitations of the current predictor/moderator analyses. As mentioned, no correction for the number of predictors/moderators was implemented, though we place greater emphasis on results significant at a .01 alpha level and on the multivariate results. Furthermore, other variables might confound the relations of the predictors/moderators examined here with outcome. The potential existence of measured or unmeasured confounding variables highlights the need to remain cautious about any causal interpretations of the findings reported herein. Moreover, the relationships reported here might be affected by the quality of the implementation of the interventions. The mediating or moderating role of the process of treatment (i.e., adherence ratings) in understanding the relation of patient variables to the effectiveness of HIV risk reduction interventions is a topic that should be explored in additional studies. Despite these limitations, the findings reported here have important practical implications for clinical implementation of these treatment and design of research studies on HIV sexual risk reduction interventions. A particular strength of this study is the use of real-world clinics and clinicians, thereby increasing the external generalizability of the findings.

## Conclusions

In summary, the predictive findings (those in monogamous relationships did more poorly) and the moderator findings (Hispanics did more poorly in the 5-session intervention) suggest that further development and testing of interventions designed to reduce unsafe sex may be warranted for certain subgroups of individuals, with particular attention to cultural sensitivity.

## Abbreviations

NIDA CTN: National Institute on Drug Abuse Clinical Trials Network; ZINB: Zero-inflated negative binomial; USO: Unprotected sexual occasions; REMAS: Real men are safe; HIV-Ed: HIV education.

## Competing interests

The authors declare that they have no competing interests.

## Authors’ contributions

PCC prepared the first draft of the manuscript. PCC, RG, DC, WT, XT, and GW designed the study (choice and definition of variables; data analytic plan). RG conducted all data analyses. All authors (RG, DC, JS, HM, WT, HH, XT, and GW) provided edits and revisions to subsequent manuscript drafts. All authors read and approved the final manuscript.
